# Supplementary material for: HIVprotI: an integrated web based platform for prediction and design of HIV proteins inhibitors
Source: J Cheminform. 2018 Mar 9;10:12. doi: 10.1186/s13321-018-0266-y (PMC5845081; doi:10.1186/s13321-018-0266-y)
Supplement: Supplementary file 2 — Additional file 2. Source code of HIVProtI web server. [file 13321_2018_266_MOESM2_ESM.zip › HIVprotI_Source-code/hivprotI/weka-3-6-6/doc/weka/gui/visualize/package-frame.html]

weka.gui.visualize


weka.gui.visualize

Interfaces 
  
*AttributePanelListener*
  
*Plot2DCompanion*
  
*PrintableHandler*
  
*VisualizePanelListener*

Classes 
  
AttributePanel
  
AttributePanelEvent
  
BMPWriter
  
ClassPanel
  
JComponentWriter
  
JPEGWriter
  
LegendPanel
  
MatrixPanel
  
Messages
  
Plot2D
  
PlotData2D
  
PNGWriter
  
PostscriptGraphics
  
PostscriptWriter
  
PrintableComponent
  
PrintablePanel
  
ThresholdVisualizePanel
  
VisualizePanel
  
VisualizePanelEvent
  
VisualizeUtils
